# Locoregional Delivery of miRNAs for Glioblastoma Treatment: A Systematic Review of Advances in Delivery Systems

**DOI:** 10.3390/pharmaceutics18040470

**Published:** 2026-04-12

**Authors:** Loganathan Chandramani Priya Dharshini, Elizaveta Gaiamova, Raphael Serreau, Emmanuel Garcion, Severine Morisset-Lopez, Patrick Baril

**Affiliations:** 1 School of Bio Sciences & Technology, Vellore Institute of Technology, Vellore 632014, India; 2 Centre de Biophysique Moléculaire (CBM), UPR 4301, UFR Sciences & Techniques, University of Orléans, 45071 Orleans, France; 3 Establishment Public De Mental Health (EPSM) Georges Daumezon, Centre Hospitalier Universitaire d’Orleans (CHUO), 45100 Orleans, France; 4 Centre de Recherche en Cancérologie et Immunologie Intégrée Nantes Angers (CRCI^2^NA), INSERM UMR 1307, CNRS UMR 6075, University of Angers, 49000 Angers, France

**Keywords:** microRNA, locoregional delivery, glioblastoma, gene delivery, therapeutic

## Abstract

Glioblastomas represent the most aggressive and lethal form of primary brain cancer and continue to pose a major challenge to global health. MicroRNAs (miRNAs), as central regulators of gene expression, are intimately involved in the initiation, progression, and therapeutic resistance of numerous malignancies, including glioblastoma. Therefore, this class of non-coding RNAs are considered to be valuable candidates for innovative therapeutic strategies. However, despite many promising preclinical studies, miRNA-based therapies have yet to be translated into routine clinical practice. In the context of glioblastoma, one of the principal obstacles to the effective delivery of synthetic miRNA therapy is their limited ability to cross the blood–brain barrier (BBB). To address this challenge, a variety of locoregional delivery strategies have been developed in recent years. In this review, we provide a detailed discussion and a state-of-the-art overview of these local delivery methods in the context of glioblastoma treatment, with a specific emphasis on their application for delivering miRNA-based therapeutic oligonucleotides, formulated either with or without synthetic nanoparticles.

## 1. Introduction

Glioblastoma (GB), the most prevalent malignant brain tumor, constitutes ~57% of gliomas and 48% of all primary malignant central nervous system (CNS) tumors [1]. This tumor type is characterized by profound heterogeneity. Multiple, distinct signaling pathways operate concurrently within a single tumor mass, creating a complex and challenging target for therapeutic intervention. Despite advancements in treatment strategies, the prognosis for GB remains poor. The average overall survival is a mere 12 to 18 months, with a 5-year survival rate falling below 6% [2].

The standard GB treatment approach includes maximal surgical resection followed by radiation therapy and chemotherapy with temozolomide (TMZ). However, it ultimately falls short due to the tumor intrinsic resistance mechanisms [3]. GB cells exhibit robust DNA repair capabilities and self-renewing properties, with glioma stem cells (GSCs) playing a pivotal role in tumor initiation and regrowth. GSCs are particularly resistant to conventional chemotherapy and radiotherapy, driving therapeutic failure and tumor relapse. In recurrent cases, the tumor invasive nature and extensive infiltration into surrounding brain regions often preclude further surgical interventions [4]. A combination of biological, molecular, and microenvironmental factors hampers effective glioblastoma therapy, resulting in persistently poor clinical outcomes. These include incomplete tumor resection, intratumoral heterogeneity, the blood–brain barrier (BBB) restricting drug delivery, and an immunosuppressive tumor microenvironment (TME) that shields cancer from the body’s defenses [5]. Each of these elements significantly contributes to treatment resistance.

**Figure 1:**
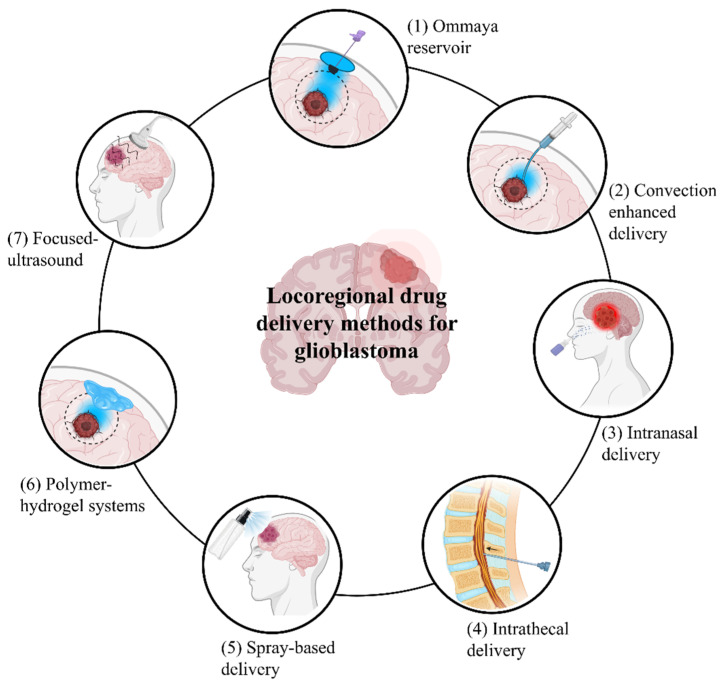
Seven distinct approaches for direct therapeutic delivery to glioblastoma are illustrated: (1) ommaya reservoir—a subcutaneous scalp-implanted dome connected to a ventricular catheter for repeated intracranial drug administration without lumbar puncture; (2) convection-enhanced delivery (CED)—stereotactically inserted cannula utilizing positive-pressure bulk flow to distribute agents beyond the tumor margin; (3) intranasal delivery—non-invasive delivery along olfactory and trigeminal pathways, bypassing the blood–brain barrier; (4) intrathecal delivery—lumbar puncture-based introduction of therapeutics into the cerebrospinal fluid for rostral intracranial distribution; (5) spray-based delivery—intraoperative direct application onto the tumor resection cavity for broad local drug deposition; (6) polymer-hydrogel systems—drug-eluting implants within the resection cavity providing sustained locoregional therapeutic release; and (7) focused ultrasound—non-invasive transcranial ultrasound for transient blood–brain barrier disruption and enhanced drug penetration (Images prepared using Microsoft PowerPoint Presentation).

Given these limitations, the development of innovative therapeutic strategies for glioblastoma has become increasingly crucial. Over the past two and a half decades, microRNAs (miRNAs) have gained significant attention in the scientific community. Initially identified between the mid-1990s and early 2000s, miRNAs were first regarded as non-functional short transcriptional products without any open reading frame and were considered as transcriptional noise. The identification of the first miRNA, lin-4, followed by let-7, as key regulators of embryogenesis in C. elegans, ultimately led to Victor Ambros and Gary Ruvkun being awarded the 2024 Nobel Prize in Physiology or Medicine. The molecular basis of miRNA activity was established earlier, in 2006, when Andrew Fire and Craig Mello were awarded the Nobel Prize for the discovery of RNA interference (RNAi)—the endogenous pathway central to miRNA-mediated gene silencing and post-transcriptional regulation. Despite extensive preclinical validation and compelling proof-of-concept studies demonstrating their therapeutic potential, miRNA-based therapies have yet to be adopted in standard medical practice. Only siRNA molecules, which exploit the RNAi pathway to downregulate specific target genes, were granted approval in 2018 by the United States Food and Drug Administration (FDA) for the treatment of hereditary transthyretin amyloidosis. In fact, a major limitation of RNAi-based therapies, as well as systemically administered drugs in general, lies in the difficulty of delivering sufficient quantities of therapeutic molecules to the tumor mass to elicit an effective antitumor response while minimizing exposure to non-tumoral healthy tissues. In this context, locoregional therapy has emerged as a promising strategy, delivering drugs directly to the tumor mass or tumor cavity post-resection. This approach concentrates therapeutic agents at the tumor site, improving efficacy against cancer cells while minimizing collateral damage to healthy cells [6]. In glioblastoma, although some bloodstream-compatible nanocarriers as dendrimers, have been developed and shown to reach brain tumors [7,8], locoregional delivery methods have proven more effective in bypassing the blood–brain barrier and enhancing drug paid load delivery These include convection-enhanced delivery (CED), intrathecal and intranasal administration, polymer-based hydrogel systems, focused ultrasound (FUS) and the optical blood–brain tumor barrier (optoBBTB) approaches to transiently open the BBB (Figure 1). All of these methods represent valid alternative strategies for glioblastoma treatment. Some have been successfully employed to deliver miRNA-based therapeutic oligonucleotides directly into glioblastoma tumors masses. In this review, we provide a comprehensive overview of locoregional approaches for the treatment of glioblastoma, describing the delivery methods and therapeutic agents employed, with a particular emphasis on their relevance and applicability to miRNA-based therapies.

## 2. Locoregional Delivery Methods for Brain Tumor

### 2.1. Catheter Delivery

#### The Ommaya Reservoir

In 1963, pioneering neurosurgeon Ayub Khan Ommaya introduced the Ommaya reservoir, a surgically implanted intraventricular device designed to facilitate repeated administration of therapeutic agents directly into the cerebrospinal fluid (CSF) [9]. The system consists of a small, dome-shaped silicone or titanium capsule connected to a fine silicone catheter, which is stereotactically positioned within one of the lateral ventricles of the brain for several days. The reservoir allows for repeated, precise intraventricular injections while minimizing the risk of infection and reducing the need for repeated lumbar punctures. It has been widely employed for the administration of chemotherapeutic agents, antibiotics, and more recently, nucleic acid-based therapies, including oligonucleotides and viral vectors, offering a reliable platform for direct CNS drug delivery in both preclinical and clinical settings [10]. A recent study suggested that administering arsenic trioxide through the Ommaya reservoir with radiotherapy post-surgery is safe and well-tolerated. The study recommends a dose of 1.5 mg for evaluation in the phase II trial [11]. To date, although the Ommaya reservoir has been successfully employed for repeated intraventricular/intrathecal administration of the therapeutic antisense oligonucleotide (ASO) nusinersen, which modulates SMN2 splicing in patients with spinal muscular atrophy, no studies have reported the use of this method for delivering miRNAs or other RNAi therapeutic oligonucleotides into central nervous system tumor tissues.

### 2.2. Convection-Enhanced Delivery

#### 2.2.1. General Considerations

Convection-enhanced delivery (CED) is a targeted regional drug delivery technique within the brain. Originally described by Bobo and colleagues in 1994, this method enables deeper and more localized drug distribution while bypassing the blood–brain barrier, which limits the efficacy of systemically administered drugs [12]. The principle relies on “bulk flow,” where positive pressure applied through an infusion catheter actively drives therapeutic agents into the interstitial spaces, overcoming the limitations of passive diffusion. This approach ensures homogeneous distribution within the surrounding parenchymal tissue and effective targeting of cells of interest [13]. Governed by Darcy’s law (v = −K∇pv = −K∇pv = −K∇p), drug velocity is proportional to both the pressure gradient (∇p∇p∇p) and the hydraulic conductivity of brain tissue (K) [14]. Optimal delivery is achieved using a pump-catheter system precisely positioned near the target tissue to maximize direct perfusion [15].

The first CED-based approach for malignant brain tumors employed a ligand-targeted toxin, transferrin-CRM107 (Tf-CRM107) [16]. Phase I clinical trials involved high-flow convection delivery through stereotactically placed catheters, resulting in tumor reduction of ≥50% in 9 of 15 evaluable patients, with minimal neurological or systemic toxicity. Subsequent Phase II trials showed a 39% response rate with 30% surviving for over a year [17]. Another notable CED-based therapy involves the direct intratumoral infusion of paclitaxel. Fifteen patients received 20 cycles of paclitaxel, resulting in a 73% response rate, albeit with transient chemical meningitis, and neurological deterioration as adverse events [18]. Additionally, Cotara^®^, a radiotherapeutic agent employing a ^131^I-labeled chimeric monoclonal antibody has been administered to malignant glioma patients, causing mild systemic effects with CNS-related adverse events such as brain edema, hemiparesis, and headache [19]. In addition to these, a variety of agents including IL13-PE38QQR, TGF-β, TP-38, topotecan, MR1-1KDEL, trabedersen, GRm13Z40-2 CTL, Delta-24-RGD, PSV-RIPO, carboplatin, muscimol, D2C7, 124I-8H9 have progressed to clinical trials for the treatment of different types of gliomas [14].

A significant limitation of CED is the rapid clearance of therapeutic drugs from the brain. Encapsulation of drugs within nanoparticles (NPs) enables extended drug retention time, thereby enhancing the efficacy of CED-based treatments [20]. Am80, a synthetic retinoic acid receptor agonist, was incorporated into polymeric micelles and delivered via CED to U87MG xenograft rats along with TMZ. This combinatorial approach significantly improved survival rates of animals compared to controls and monotherapy [21]. In another study, nanoparticles were generated using a non-emulsion technique to incorporate iron oxide into the nanoparticle shell and to load temozolomide (TMZ) as the therapeutic payload [22]. The presence of iron oxide allows precise, non-invasive visualization of nanoparticle biodistribution and intratumoral uptake after administration via convection-enhanced delivery (CED). Serial T2-weighted MR images from a representative scan reveal a hypointense signal centered at the CED infusion site, whereas bolus injection of the same formulation fails to achieve ventral distribution of nanoparticles beyond the catheter tip and instead results in backflow along the catheter tract. This highlights the importance of continuous low-pressure infusion through the catheter by CED to ensure optimal tissue distribution. Notably, CED of TMZ-bearing nanoparticles prolongs the survival of animals with intracranial xenografts compared to controls [22]. In another strategy, chitosan lipid nanocapsules co-encapsulating siRNAs targeting both the epidermal growth factor receptor (EGFR) and galectin were evaluated in an orthotopic glioblastoma mouse model following convection-enhanced delivery (CED). This approach significantly improved animal survival compared with nanocapsules loaded with siRNA alone or temozolomide (TMZ, 40 mg/kg) alone. Consistent with these findings, a marked downregulation of EGFR and galectin-1 protein expression in tumor tissues was observed [23].

**Figure 2:**
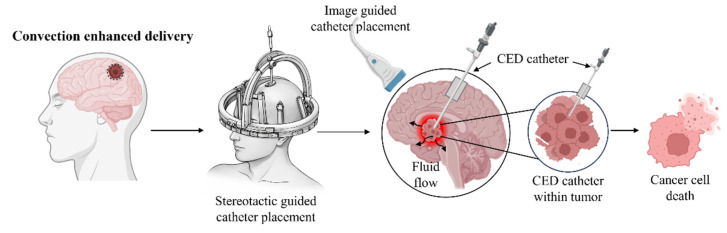
Illustration showing convection-enhanced delivery of druggable molecules within tumor cells resulting in localized therapeutic treatment. CED involves stereotactic-guided placement of a catheter into the brain tumor, followed by image-guided confirmation of catheter positioning. Positive-pressure infusion drives bulk fluid flow directly through the tumor interstitium, enabling localized distribution of therapeutic agents. This approach bypasses the blood–brain barrier and results in targeted cancer cell death (Image prepared using Microsoft Powerpoint Presentation).

The application of CED with NPs to treat glioma or glioblastoma in clinical translation has significantly progressed. A single CED administration of irinotecan-containing nanoliposomal CPT-11 and Doxil were found to be accumulated in the CNS for more than 36 days, highlighting combinatorial therapy over monotherapy [24]. Panobinostat-encapsulated gold nanoparticles, MTX110 (ClinicalTrials.gov ID: NCT03566199), have been investigated for their efficacy in patients with diffuse intrinsic pontine glioma (DIPG). MTX110 demonstrated potent antitumor activity and improved survival in DIPG models, with an IC_50_ comparable to free panobinostat but without toxicity. Its mechanism of action involves G1 phase cell cycle arrest and a reduction in pyruvate-to-lactate conversion, and a tissue half-life of approximately 2 h [25]. The workflow of convection-enhanced delivery is depicted in Figure 2.

#### 2.2.2. Current Advances in Delivering miRNAs Using Convection-Enhanced Delivery (CED) Within GBM Tumors and/or Throughout the Central Nervous System

The first preclinical study investigating CED for miRNA delivery in glioblastoma models was conducted approximately ten years ago [26]. In this pioneering work, the authors provided experimental evidence that delivery of a 2′-O-methoxyethyl anti-miRNA let-7 oligonucleotide (AMO), incorporating a combined phosphodiester/phosphorothioate backbone, into glioblastoma tumors via CED is both feasible and functional. They showed that repeated administration of this anti-let-7 miRNA AMO over one month was well tolerated, retained anti-miRNA activity, and achieved robust target de-repression of HMGA2 mRNA. As with other early studies in the miRNA field at that time, the authors did not assess the therapeutic efficacy of anti-let-7 miRNA. Instead, they demonstrated that the concept of CED is viable for delivering anti-miRNA molecules. Let-7 was chosen as a target due to its status as one of the most extensively characterized microRNAs, rather than for its direct therapeutic relevance.

It was only a year later that the therapeutic efficacy of another AMO therapy delivered via CED was assessed, using miR-10b as the therapeutic target. miR-10b is a well-characterized oncogenic miRNA, highly expressed in several tumor types, including GBM subtypes [27] and known to regulate the cell cycle through MBNL1–3, SART3, and RSRC1 mRNA targets [28]. Delivery of anti-miR-10b AMOs via CED effectively restored target expression and markedly reduced the growth and progression of established intracranial GBM. Notably, and somewhat unexpectedly, systemic administration of anti-miR-10b AMOs also led to mRNA de-repression and a reduction in tumor burden. However detectable levels of anti-miR-10b AMO in the liver and lungs were observed in contrast to the localized delivery achieved through CED. Nevertheless, no significant systemic toxicity was observed following AMO administration by either local or systemic routes [29].

In another study, the delivery of a therapeutic oligonucleotide targeting a miRNA was evaluated after complexation with 2 different nanoparticles for infusion into the GBM tumor via CED [30]. The AMO used were directed against the well-known oncogenic miRNA 21 [31]. The delivery systems encountered a cationic poly(amine-co-ester) polymer called PACE while the second consisted of a block copolymer of poly(lactic acid) and hyperbranched polyglycerol (PLA-HPG). Both systems supported more or less equal intracellular uptake and miRNA-21 suppression, which in turn elevated PTEN expression and promoted apoptosis in human GBM cells. In vivo, when delivered via CED into intracranially implanted gliomas, these two delivery platforms achieved effective local reduction in miRNA-21, upregulated pro-apoptotic gene expression, and enhanced responsiveness to chemotherapy, ultimately resulting in prolonged survival of the animals. However, when administered alone without temozolomide, only a modest or no significant reduction in tumor burden was observed, indicating that AMO therapy targeting miRNA-21, despite it being a potent oncogenic driver, is insufficient on its own to halt tumor progression.

More recently, we exploited our miR-ON RILES system to precisely characterize the kinetics of miRNA-mediated mRNA silencing in a preclinical glioblastoma model following CED infusion of synthetic miRNA oligonucleotides [32]. We showed that, despite locoregional delivery into tumor masses, miRNA oligonucleotides distribution was heterogeneous across individual tumors yielding distinct silencing dynamics—ranging from rapid and efficient responses in some tumors to slower but more sustained effects in others. We further showed that two intratumoral infusions by CED of a miRNA oligonucleotide were sufficient to sustain a prolonged gene-silencing effect lasting up to 16 days. Based on this finding, we optimized both the dose and timing of a delivery protocol for the administration of miRNA-200 mimic oligonucleotides. This miRNA was selected because our gain-of-function studies demonstrated that miR-200c transfection markedly inhibited tumor cell proliferation by inducing G1 cell-cycle arrest and also reduced cell migration through deregulation of the transcription factors ZEB1 and ZEB2, as well as Vimentin and E-cadherin expression. However, no significant induction of tumor apoptosis or cell death was observed. Nevertheless, when miR-200c mimic oligonucleotides complexed with a lipopolyplex formulation were infused into the GBM tumor masses by CED, a significant delay in tumor growth was observed but this effect was transient and reversible [33]. Consistently, Kaplan–Meier survival analysis revealed no statistically significant differences between treated and control groups, although the median survival of miR-200c-treated animals was prolonged by approximately two days compared with controls. These data, as observed for miRNA-21, underscore the challenge of inhibiting glioblastoma tumor growth without concomitant chemotherapy, reflecting the highly aggressive nature of this tumor type.

Most recently, an innovative dual anti-miRNA strategy was evaluated across two GBM mouse models. This approach employed short (8-mer) γ-modified peptide nucleic acids (sγPNAs) designed to target the seed regions of the oncomiRs miRNA-10b and miRNA-21. These sγPNAs were formulated with the aforementioned aldehyde-functionalized PLA-HPG block copolymer enabling enhanced transfection efficiency and tumor-selective uptake. When combined with temozolomide and delivered via CED, the sγPNA nanoparticles produced a remarkable therapeutic benefit, extending survival of mice to over 120 days in a preclinical GBM PDX model [34]. During autopsy, robust target engagement was observed, with 72% knockdown of miRNA-10b and 95% suppression of miRNA-21, accompanied by coordinated regulation of ten downstream genes identified through RNA-seq, including PDGFRA, PDGFRB, VEGFA, ITGB8, ITGA10, ITGA11, ANGPT1, ANGPT2, PRKCA, and IL-6. Collectively, these findings suggest that integrating anti-miR sγPNA nanoparticles into the current temozolomide-based standard of care for GBM may provide a promising strategy to enhance therapeutic outcomes.

In recent years, an increasing number of preclinical studies have explored miRNA delivery using CED for glioblastoma therapy (Table 1), raising the expectation that clinical trials may follow in the future.

### 2.3. Intranasal Drug Delivery

#### 2.3.1. General Considerations

**Figure 3:**
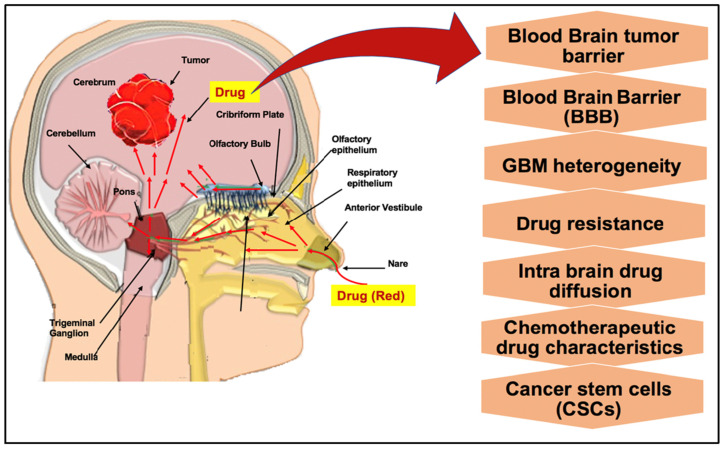
Intranasal drug delivery [43]. Schematic representation of the intranasal route for drug delivery to the brain, highlighting pathways that bypass the blood–brain barrier (BBB). Following administration into the nasal cavity, druggable molecules (red arrows) are absorbed across the olfactory and respiratory epithelia. From the olfactory region, compounds can cross the cribriform plate and reach the olfactory bulb via intracellular axonal transport or extracellular perineural diffusion along olfactory neurons. In parallel, druggable molecules deposited in the respiratory epithelium can access the trigeminal nerve branches, enabling transport toward both anterior and posterior brain regions, including the brainstem. Additional distribution may occur via perivascular spaces and cerebrospinal fluid, facilitating wider intra-brain diffusion. This direct nose-to-brain delivery circumvents the BBB and the blood–brain tumor barrier, improving drug access to intracranial tumors such as glioblastoma (GBM). The figure also highlights key therapeutic challenges, including GBM heterogeneity, drug resistance, limited intra-brain diffusion, chemotherapeutic constraints, and the presence of cancer stem cells, all of which impact treatment efficacy.

Intranasal transport refers to the direct delivery of therapeutic agents from the nasal cavity to the brain (Figure 3). The main advantage of intranasal delivery over parenteral administration is that it avoids elimination by the liver, gastrointestinal tract, and kidney filtration as well as serum degradation. After intranasal administration, the nasal mucosa serves as an effective absorption site for drug transport across the epithelial barrier via two main pathways: extracellular (extrinsic) and intracellular (intrinsic) routes. The extracellular pathway, which represents the predominant mechanism for nose-to-brain delivery, involves paracellular diffusion or bulk flow through the intercellular spaces of the nasal epithelium, allowing direct access to the central nervous system while bypassing the blood–brain barrier. In contrast, the intracellular pathway relies on endocytosis of therapeutic agents into olfactory sensory neurons and trigeminal nerve endings, followed by axonal transport toward the brain. Although generally less efficient than extracellular transport, this intrinsic pathway enables highly specific neuronal delivery [35].

Intranasal administration has been widely used in clinical practice for decades to deliver drugs that are poorly suited for oral administration, including corticosteroids, decongestants, antihistamines, and vaccines, highlighting its safety, feasibility, and translational potential for central nervous system targeting [36].

At the end of the last century the potential of intranasal administration for targeting the central nervous system (CNS) has garnered significant scientific interest [37,38,39]. Nowadays, intranasal drug delivery has emerged as a promising strategy for glioblastoma treatment, enabling the administration of a wide range of therapeutic agents, including conventional chemotherapeutic drugs, oligonucleotides, oncolytic viruses, and even neural stem and progenitor cells (NSPCs). For instance, Wang et al. (2003) demonstrated the feasibility of intranasal methotrexate delivery to the central nervous system [40]. In this study, intranasal administration resulted in higher methotrexate concentrations in the cerebrospinal fluid (CSF) and lower systemic exposure in plasma compared with intravenous administration, highlighting the advantage of this route for targeted brain delivery. In a second example, Hashizume et al. (2008) reported the successful intranasal delivery of the telomerase inhibitor GRN163, a FITC-labeled oligonucleotide, via the nose-to-brain pathway [41]. Their findings provided clear evidence that oligonucleotide-based therapeutics can effectively bypass the blood–brain barrier following intranasal administration and reach brain tissues relevant for glioblastoma therapy. Of note, FITC-labeled GRN163 was readily detected within tumor cells, with intracellular accumulation reaching a peak approximately 4 h after intranasal administration. Moreover, repeated daily intranasal delivery of GRN163 over a 12-day treatment period led to a marked improvement in the survival of treated animals, with the median survival time of 35 days in the control group and to 75.5 days in animals treated with GRN163.

Notably, intranasal drug delivery is sufficiently versatile to allow the administration of even cellular therapies. In this context, Reitz et al. (2012) investigated the feasibility of intranasal delivery of neural stem/progenitor cells (NSPCs) and demonstrated that intranasal administration enabled rapid and targeted migration of these cells toward intracerebral gliomas [42].

#### 2.3.2. Current Advances in Delivering miRNAs by Intranasal Drug Delivery in GBM Tumors and/or Throughout the Central Nervous System

One of the earliest studies employed an intranasal delivery approach to transport a combination of anti-miR-21 antisense oligonucleotides (AMOs) and miR-100 mimic oligonucleotides. This strategy enabled the delivery of these miRNA therapeutics to implanted glioblastoma tumors. These miRNA therapeutics were formulated with a polyfunctional gold–iron oxide nanoparticle coated with an α-cyclodextrin–chitosan (CD-CS) hybrid polymer to ensure stability and efficient uptake by the nasal epithelium. The nanoparticles were further functionalized with a PEG–T7 peptide for efficient targeting to GBM cells [44]. The resulting polyGION nanoparticles were spherical in shape, nearly neutral in charge, and approximately 50 nm in size. These NP were efficiently taken up by glioblastoma cells in cellulo. Notably, intranasal administration of T7-targeted polyGIONs led to robust accumulation of Cy5-labeled miRNAs within glioblastoma tumors in treated mice, compared with nanoparticles lacking the T7 targeting moiety. From a therapeutic point of view, these T7-targeted polyGIONs alone were insufficient to confer a significant therapeutic benefit; however, when combined with systemic delivery of temozolomide (TMZ), a clear therapeutic advantage was observed. Mice receiving T7-targeted polyGIONs loaded with anti-miR-21 and miR-100 in combination with TMZ survived up to 44 days, compared with 32 days for TMZ alone, 30 days for anti-miR-21 plus miR-100 without vectorization, and 16 days for untreated controls.

In another study, Ha et al., 2021, evaluated a dual-targeting strategy for glioblastoma focusing on the receptor for advanced glycation end-products (RAGE), which is frequently upregulated in GBM, as the primary therapeutic target, and the oncomiRNA miR-21 as a secondary target [45]. The authors demonstrated that RAGE-antagonist peptides (RAPs) could self-assemble with cholesterol-conjugated anti-miR-21 antisense oligonucleotides (antagomiR-21) to form nanoparticles without the need for additional carriers. These antagomiR-21/RAP nanoparticles were administered intranasally in a preclinical glioblastoma animal model. A significant reduction in miRNA-21 expression was observed within tumor masses, which correlated with increased expression of the pro-apoptotic genes PTEN and PDCD4 and the induction of tumor cell apoptosis. Notably, decreased expression of vascular endothelial growth factor (VEGF) was also detected within the tumor and was associated with a reduction in CD31-positive endothelial cells. This latter effect was attributed to the activity of the RAPs. However, despite these molecular and cellular effects, no significant therapeutic benefit was observed when the nanoparticles were administered alone, in the absence of concomitant chemotherapeutic treatment.

As an alternative to synthetic nanoparticles, extracellular vesicles (EVs) can be exploited for the intranasal delivery of miRNA oligonucleotides to glioblastoma. In a study by Wang et al., 2021, neural stem cells were engineered in cellulo to overexpress the C-X-C chemokine receptor type 4 (CXCR4), enabling the production of EVs displaying CXCR4 on their surface [46]. This modification facilitated targeted binding to stromal-derived factor-1 (SDF-1), overexpressed in glioblastoma tumors. The authors further developed a scalable microfluidic platform to efficiently load miRNAs therapeutic (anti-miR-21 AMO and miR-100 mimic oligonucleotides) into the engineered CXCR4-EVs. The resulting miRNA-loaded EVs exhibited uniform size and achieved markedly higher (approximately one order of magnitude) miRNA loading efficiency and cellular uptake in GBM cells compared with conventional transfection methods. Notably, intranasal administration of miRNA-loaded CXCR4-EVs in mice led to efficient accumulation of anti-miR-21 and miR-100 within implanted glioblastomas and significantly improved therapeutic outcomes when combined with temozolomide (TMZ). Specifically, mice treated with CXCR4-engineered EVs carrying anti-miR-21 and miR-100 in combination with TMZ exhibited a median survival of 48 days, compared with 21 days for untreated controls, 28 days for non-engineered EVs plus TMZ, and 38 days for non-engineered EVs carrying miR-21/miR-100 plus TMZ. This study provides the first preclinical validation of engineered extracellular vesicles as effective intranasal miRNA delivery vehicles for GBM therapy and highlights as well the translational potential of microfluidics-based miRNA encapsulation coupled with a non-invasive nose-to-brain delivery strategy.

These first studies (Table 2) that used intranasal microRNA delivery for glioblastoma treatment provide a strong foundation for further investigation of this highly patient-compliant drug administration method.

### 2.4. Intrathecal Drug Delivery

#### 2.4.1. General Considerations

Intrathecal (IT) delivery refers to the direct administration of therapeutic agents into the cerebrospinal fluid (CSF) within the subarachnoid space, bypassing the blood–brain barrier (BBB) and allowing for direct access to the central nervous system (CNS) [47]. This route provides high local drug concentrations in the CSF and adjacent neural tissues while minimizing systemic exposure and off-target effects. IT delivery has been extensively used for a range of therapeutics, including chemotherapeutics, antisense oligonucleotides, viral vectors, and analgesic agents. The technique can be performed via lumbar puncture or through implanted intrathecal catheters and pumps, enabling either single or repeated administrations. Distribution within the CSF is influenced by factors such as molecular size, lipophilicity, CSF flow dynamics, and the presence of active transport mechanisms. While IT delivery offers distinct advantages for targeting CNS malignancies or genetic disorders affecting the brain and spinal cord, it also requires careful consideration of potential risks, including infection, neurotoxicity, and procedural complications [48].

**Figure 4:**
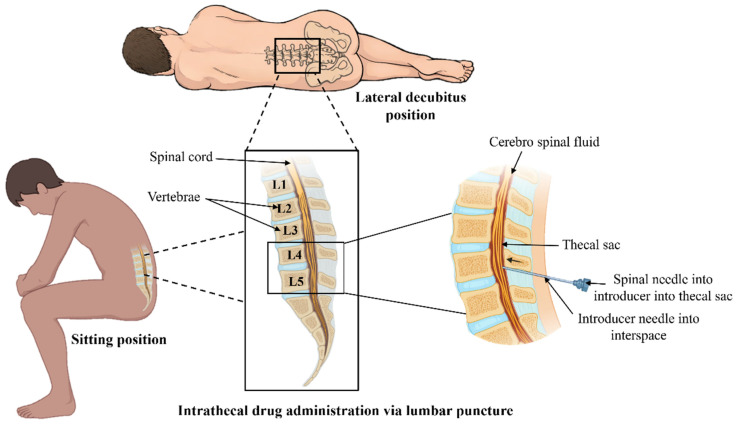
Intrathecal drug delivery via lumbar puncture for glioblastoma: Patient positioning in either lateral decubitus or sitting (fetal) posture maximizes spinal flexion to widen interspinous spaces. The intercristal line identifies the L4 vertebral level, guiding needle insertion at L3–L4 or L4–L5 interspaces- below the conus medullaris to prevent spinal cord injury. The needle traverses the skin, subcutaneous tissue, spinal ligaments, epidural space, and dura-arachnoid complex to access the subarachnoid space, allowing intrathecal administration of therapeutic agents into the cerebrospinal fluid for rostral distribution toward the intracranial tumor (Image prepared using Microsoft PowerPoint Presentation).

In GBM-associated neoplastic meningitis rodent models, intrathecal administration of an oncolytic poliovirus PVS-RIPO showed no clinical or histological toxicity. Treated rats demonstrated increased median survival, with a subset achieving long-term survival and complete tumor remission [49]. Intrathecal delivery could be particularly beneficial for oncolytic virus therapy, as it protects the virus from the host immune system, enabling more efficient infection and spread within the tumor. Intrathecal injection of methotrexate (MTX)-loaded nanocapsules in GB-induced rat models demonstrated immunomodulatory effects, upregulating the CD73 enzyme. This enzyme increase facilitates elevated adenosine production and fosters an immunosuppressive microenvironment [50]. In humans, intrathecal bevacizumab delivery for leptomeningeal spread (LMS) from recurrent GB demonstrated its potential safety and feasibility. This study paves the way for dose-escalation trials to optimize therapeutic outcomes [51]. The illustration of intrathecal drug delivery is given in Figure 4.

#### 2.4.2. Current Advances in Delivering miRNAs by Intrathecal Drug Delivery in GBM Tumors and/or Throughout the Central Nervous System

Several studies have reported successful intrathecal miRNA delivery to the brain in preclinical in vivo models but only a limited number have investigated this approach in the context of glioblastoma treatment.

Outside the glioblastoma field, Wallach et al., (2021) employed intrathecal administration of a panel of miRNA oligonucleotides selected based on their upregulation in several central nervous system (CNS) disorders [52]. The objective was to identify miRNAs capable of functioning as Toll-like receptor (TLR) ligands and to determine which of them might contribute to neurodegeneration and microglial accumulation in the cerebral cortex of naïve mice. Among the 12 miRNAs examined, 6 were found to activate human TLR7 and/or TLR8 in human-derived macrophages and murine microglia. Very surprisingly, no strict correlation was observed between TLR activation and the presence of GU-rich sequences or specific secondary structural features in the miRNA oligonucleotides. This finding suggests that TLR-mediated immune responses to miRNAs cannot be reliably predicted solely based on sequence motifs or predicted folding patterns, emphasizing the critical importance of carefully considering potential off-target immune effects when designing therapeutic oligonucleotides for intrathecal administration to treat CNS disorders, including glioblastoma. In another study, Barbosa et al., (2022) reported that intrathecal delivery of a motor neuron-derived secretome, previously treated with anti-miR-124, was able to prevent early-stage neurodegenerative progression in an amyotrophic lateral sclerosis (ALS) model [53]. Additionally, Huang et al., (2019)**,** employed intrathecal administration of a miR-873a-5p antagomir to investigate the role of this miRNA in the development of morphine tolerance [54]. Furthermore, the intrathecal route has already been applied in a Phase I clinical trial for bivalent CAR-T cells targeting EGFR and IL13Rα2 in glioblastoma patients [55].

In the context of glioblastoma treatment, a comparative study evaluating intratumoral (ITu), intraventricular (ICV), and intrathecal (IT) delivery of AMOs demonstrated that the intrathecal route was less effective than the other approaches [56]. Following administration of anti-Let-7 AMO, increased expression of Let-7 target genes, including HMGA2, LIN28B, and IGFBP2, was observed in mice treated via ITu or ICV delivery, whereas intrathecal injection produced no significant changes. Notably, only intratumoral delivery of anti-Let-7 AMO via convection-enhanced delivery (CED) resulted in upregulation of these targets in both the tumor core and peripheral tumor regions. Overall, intratumoral delivery proved to be the most efficient route for achieving effective anti-Let-7 delivery to the tumor and adjacent brain tissue, while ICV administration provided broader but comparatively limited efficacy, and intrathecal delivery remained largely ineffective.

### 2.5. Spray-Based Delivery

Another locoregional strategy, referred to as spray-based delivery, employs a custom-designed, mechanically engineered spray device optimized for safe intraoperative use, enabling the aerosolized administration of multiple classes of drugs [57,58]. This system incorporates bioadhesive pectin hydrogels and polylactic acid–polyethylene glycol-coated drug nanocrystals, which can be dispersed as volatile microdroplets. In a study by McCrorie et al., 2020, the authors evaluated a sprayable hydrogel strategy for localized delivery of Etoposide and Olaparib nanocrystals (NCPPs) into brain tissue adjacent to surgical cavities [59]. They repurposed pectin, demonstrating its biocompatibility, bioadhesion, and gelling at physiological brain calcium levels. Etoposide and olaparib chemotherapeutics were selected for coating with polylactic acid–polyethylene glycol (PLA–PEG) to generate PLA–PEG-coated drug nanocrystals. This drug encapsulation strategy involves the initial formation of drug nanocrystals, followed by polymer coating, resulting in nanoparticles with high drug loading. Nanocrystal formation occurs when drug solubility is exceeded, inducing nucleation and subsequent growth through Ostwald ripening. The PLA–PEG coating provides steric stabilization, limiting aggregation and enhancing colloidal stability [60]. In vivo experiments confirmed effective distribution of Cy5-labeled PLA–PEG-coated drug nanocrystals in tissue surrounding the resection site, with persistence for up to 14 days. Moreover, no local inflammatory response was observed in sprayed tissues at mouse autopsy. Overall, this work highlights the preclinical development of a novel locoregional spray delivery system suitable for intracranial surgical models of malignant brain tumors. So far, this method has not been explored for the delivery of miRNAs or other therapeutic oligonucleotides into tumor tissue.

### 2.6. Polymeric Hydrogel Systems

#### 2.6.1. General Considerations

Hydrogels are three-dimensional, crosslinked networks of hydrophilic polymers that form viscoelastic, highly hydrated matrices capable of mimicking the extracellular environment of biological tissues. The chemical composition, crosslinking density, and molecular architecture of the polymers determine hydrogel bioadhesivity, biocompatibility, degradation kinetics, and diffusional properties. These tunable parameters enable precise control over drug encapsulation, protection, and spatiotemporal release profiles, making hydrogels particularly attractive platforms for sustained and localized drug delivery applications [61,62,63]. Polymer hydrogel systems are locally delivered via intratumoral injection or implantable constructs, each tailored for specific therapeutic applications. Injectable hydrogels undergo a rapid sol–gel transition at physiological temperatures, forming a deep intracortical drug reservoir. This configuration allows sustained drug release and targeting of residual tumor cells. Another type of implantable hydrogel is designed for direct placement into the resection cavity, where it not only enables localized therapeutic delivery but also promotes wound healing and tissue regeneration through the incorporation of bioactive agents [64].

In a study by de la Puente et al., (2018), an injectable chitosan hydrogel was designed to deliver TMZ and ^131^I directly into the resection cavity for localized chemo-radiotherapy of GBM [65]. The hydrogel exhibited sustained retention of ^131^I with negligible release over 42 days, confining ^131^I to the tumor site with minimal dissemination. TMZ was released within 48 h, achieving 10-fold higher tumor concentrations than systemic delivery in vivo, leading to tumor reduction and improved survival rates. A bioinspired green hydrogel (BVSF), integrating biliverdin into a silk fibroin matrix, was developed and injected intratumorally for anti-glioma photothermal therapy and wound healing in C6 tumor-bearing mice. Under near-infrared (NIR) irradiation, it exhibited tunable photothermal properties, effectively ablating glioma cells and promoting wound healing with anti-inflammatory and pro-angiogenic effects in vitro and in vivo [66]. Wang et al. (2022) developed a thermosensitive hydrogel incorporating glioma-homing peptide-modified paclitaxel-loaded NPs (PNP_PTX_) and mannitolated immunoadjuvant CpG-loaded NPs (MNP_CpG_) [67]. After being injected into the post-surgical tumor cavity, the hydrogel self-assembled into a gel-drug reservoir for sustained PNP_PTX_ release, targeting residual glioma cells and promoting tumor antigen production. Concurrently, MNP_CpG_ enhances antigen presentation, activating CD^8+^ T cells and natural killer (NK) cells, reversing the TME and preventing recurrence. A dual-sensitive hydrogel loaded with bis(2-chloroethyl) nitrosourea (BCNU) and TMZ was developed to respond to temperature and redox potential. After being injected into the resection cavity, this system significantly improved outcomes, achieving a median survival of 65 days, which was double that of the surgery-only group [68].

In recent research, Caraffi et al. (2025) presented hybrid polymeric scaffolds that can be injected or implanted into the brain after tumor resection [69]. These scaffolds act as delivery platforms for drugs, biologics, or combination therapies, enabling controlled, localized release at therapeutically relevant concentrations. By avoiding systemic circulation and bypassing the BBB, scaffolds enhance local drug levels. Concentrating therapies at the tumor site reduces off-target side effects compared with systemic administration. Scaffolds can co-deliver agents such as chemotherapy, nanoparticles, and cell-based therapies (e.g., CAR-T cells) for synergistic effects. Scaffold design integrates biocompatible polymers engineered to match mechanical and degradation properties suitable for brain tissue.

#### 2.6.2. Current Advances in Delivering miRNAs Using Polymeric Hydrogel Systems in GBM Tumors and/or Throughout the Central Nervous System

To date, as mentioned above, hydrogels have been extensively studied as matrices for the localized delivery of therapeutic agents, primarily conventional chemotherapeutic drugs. However, only a few studies have explored the use of injectable hydrogel platforms for miRNA delivery in glioblastoma [70]. A slightly larger number of studies have focused on nanogels. While hydrogels are macroscopic or microscopic polymeric networks mainly employed as local drug depots, nanogels are nanoscale, particulate hydrogels that can serve as carrier systems for systemic administration, facilitating cellular uptake and efficient gene delivery. Early examples of miRNA-based nanogel systems include the work of Shatsberg et al. (2016) [71]. The authors developed polymeric nanogels (NGs) based on a polyglycerol scaffold for miRNA delivery in glioblastoma (GBM) therapy, focusing on the tumor-suppressive miR-34a. Treatment of U-87 MG GBM cells with NG-miR-34a polyplexes led to downregulation of miR-34a target genes, inducing apoptosis, cell cycle arrest, and inhibition of proliferation and migration. Notably, NG-miR-34a administration in an orthotopic animal model of glioblastoma significantly inhibited tumor growth as compared to control treatments. Liu et al. encapsulated anti-miR-21 AMO within a pH-responsive polymeric nanogel formed by in situ polymerization, enabling stability under physiological conditions and miRNA oligonucleotide release in the acidic endosomal environment of ovarian cancer cells. In general miRNA loading in nanogels is generally achieved through electrostatic self-assembly between cationic polymer groups and anionic nucleic acids [72]. However, Dispenza et al. (2017) reported a covalent conjugation of anti-miR-31 AMO to a poly(N-vinyl pyrrolidone)-based nanogel without loss of biological activity. Notably, several miRNA delivery platforms rely on redox-sensitive nanogels crosslinked by disulfide bonds, allowing intracellular degradation and cargo release [73].

### 2.7. Focused Ultrasound (FUS) and BBB Opening

#### 2.7.1. General Considerations

Focused ultrasound (FUS) technology enables the targeted delivery of focused ultrasound waves to specific regions of the brain, selectively targeting tumor cells while minimizing adverse effects on surrounding healthy tissue. The applications of FUS technology are diverse, encompassing high-intensity thermal ablation of neoplastic tissue as well as low-intensity, transient disruption of the blood–brain barrier to facilitate improved therapeutic delivery to tumor sites [74].

FUS in combination with microbubbles (MBs, ultrasound contrast agents) has been shown to induce localized and reversible blood–brain barrier (BBB) opening without causing damage to the brain parenchyma in various preclinical models, including rodents and non-human primates [75,76]. Using FUS with microbubbles for BBB opening, Hong-Jian Wei et al. (2021) enhanced etoposide delivery to glioblastoma tumors in mice [77]. Etoposide is an antimetastatic agent with anticancer activity. While it showed good efficacy in vitro, its effect in vivo was limited due to poor brain penetration using conventional administration methods. The authors observed a significant increase in survival when etoposide was delivered with FUS and MBs (median survival: 25 days) compared to the control, etoposide alone, and FUS alone groups (median survival: 19 days).

In a preclinical study, Wei et al. (2013) tested FUS for enhancing the efficacy of temozolomide (TMZ) against glioblastoma in vivo. They demonstrated that the mean survival time increased to 26.3 ± 8.0 days, representing a 37.7% increase compared to the control group, but this effect was observed only for the TMZ + FUS group. Moreover, increasing the TMZ concentration did not further improve survival [78].

Magnetic resonance imaging-guided focused ultrasound (MRI-guided FUS, MRgFUS) has been developed to enhance the penetration of nanoparticles (NPs) into the brain by transiently disrupting the tight junctions between endothelial cells in the blood–brain barrier (BBB). When microbubbles, injected systemically, reach the focal point of the ultrasound wave, they undergo cavitation, inducing oscillations that create cavities large enough to allow systemic NPs to pass through the barrier. This effect can persist for up to 6–8 h, leading to a temporary disruption of the BBB [79]. The main difference between MRgFUS and FUS + MBs is that MRgFUS uses MRI-based real-time targeting, allowing higher precision and real-time monitoring of BBB opening or temperature.

In 2021, Anastasiadis et al. (2021) reported results from a Phase 0 clinical trial investigating the use of MRgFUS to achieve BBB opening in patients with infiltrating gliomas. They found that MRgFUS produced safe, reproducible, and spatially controlled BBB openings through the intact skull, and that microbubble acoustic emission signals could be used to titrate and monitor BBB disruption in real time. Importantly, treatment significantly increased tracer accumulation in targeted tumor regions, suggesting improved potential for drug delivery into glioma tissue. These findings highlight the potential of this method to enhance delivery of therapeutics and improve treatment strategies for brain tumors [80].

#### 2.7.2. Current Advances in Delivering miRNAs Using Focused Ultrasound (FUS) and BBB Opening GBM Tumors and/or Throughout the Central Nervous System

Recent advances in ultrasound-mediated delivery strategies have opened promising avenues for enhancing RNAi therapeutic and RNA transport across the blood–brain barrier (BBB) in glioma and glioblastoma. Initially, the delivery of shRNA and siRNA using focused ultrasound (FUS) was demonstrated to be both feasible and effective.

In 2018, Guanjian Zhao et al. presented new polymer formulation which can deliver successfully shRNA to glioblastoma in rat models in combination with FUS. Survival was almost two times higher by FUS + MB-shBirc5-lipo-NGR treatment compared to control and almost 1.5 times higher compared to MB-shBirc5-lipo-NGR treatment with no FUS. They concluded that their polymer with shRNA in combination with FUS significantly delayed tumor growth and increased rat survival more than the use of either treatment alone [81]. Later Yutong Guo et al. used cationic nanoparticles combined with microbubble-enhanced ultrasound to deliver siRNA to glioma in mouse model. They obtained significant uptake increase in Cy5-siRNA in tumors treated by FUS compared to no FUS treatment [82]. In recent studies, Anh Duy Do et al. (2025) reported that exosome used in combination with MB-FUS for siRNA delivery to medulloblastoma and glioma preclinical models [83]. They used fluorescently labeled lipid-polymer hybrid (LPH) nanoparticle loaded with a test siRNA (RohB-LPH loaded with Cy5-siRNA, LPH:Cy5-siRNA) and a therapeutic Smoothened (SMO) targeting siRNA (LPH:SMO-siRNA). The intensity of fluorescence in mouse brain was threefold higher for FUS treated than that of mice without FUS. Moreover, they demonstrated significant increase in survival compared to control group [82].

The same approach was also evaluated for miRNA delivery in GBM by FUS. Zhan et al. demonstrated that focused ultrasound (FUS) could transiently enhance BBB permeability, allowing miR-1208-loaded exosomes to accumulate in glioma tissue and, as a result, inhibit malignant progression through modulation of METTL3 and TGF-β signaling pathways [84]. Earlier work in abstract conference by Vega et al. introduced MR-guided FUS for the delivery of polymeric, brain-penetrating nanoparticle–miRNA conjugates, demonstrating proof-of-concept for image-guided, spatially controlled RNA delivery to glioblastoma [85]. More recently, Shumer-Elbaz et al. leveraged low-frequency FUS combined with microbubbles to facilitate delivery of lipid nanoparticle (LNP) RNA formulations across the BBB into glioblastoma. This approach represents a significant step toward clinically translatable RNA therapeutics, achieving enhanced intratumoral RNA accumulation without apparent neurotoxicity [86].

Currently, studies on miRNA delivery using focused ultrasound (FUS) remain limited, likely due to the recent development of this approach. Nevertheless, this strategy is promising, primarily because, as noted above, it offers a non-invasive locoregional delivery method that can be applied preoperatively—unlike convection-enhanced delivery (CED), spray-based methods, or polymeric hydrogel implantation. It also presents a lower risk than intrathecal injection and demonstrates superior bioavailability compared to intranasal drug delivery (Table 3).

### 2.8. Optical Blood–Brain-Tumor Barrier (optoBBTB)

**Figure 5:**
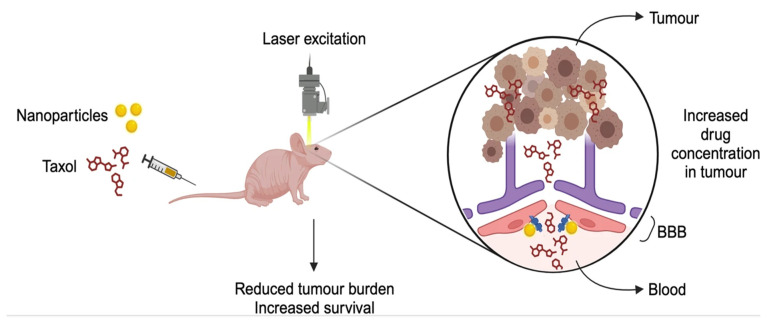
OptoBBTB [90]. Schematic illustration of the optically mediated blood–brain tumor barrier (OptoBBTB) opening strategy to enhance drug delivery to brain tumors. The approach relies on targeted vascular modulation, typically involving light-activated nanoparticles or photosensitive agents localized at the tumor vasculature. Upon light stimulation, transient and localized disruption of endothelial junctions occurs, increasing vascular permeability specifically within the tumor region. This controlled opening facilitates enhanced penetration of therapeutic agents from the bloodstream into the tumor parenchyma, overcoming limitations imposed by the blood–brain barrier (BBB) and the blood–brain tumor barrier (BBTB). The figure highlights the spatial and temporal precision of OptoBBTB, allowing selective drug delivery while minimizing off-target effects in healthy brain tissue. Key parameters influencing this process include light exposure conditions, nanoparticle targeting efficiency, and vascular heterogeneity within the tumor microenvironment.

Very recently, a novel locoregional delivery strategy called optical blood–brain tumor barrier (optoBBTB) has been developed. This approach is based on the use of gold nanoparticles engineered to specifically target the tight junction protein JAM-A expressed by endothelial cells of the blood–brain barrier (BBB). These nanoparticles were administered intravenously in a mouse model of glioblastoma together with a chemotherapeutic agent, such as paclitaxel (Taxol) [87]. Following systemic administration, a transcranial picosecond laser at 532 nm is applied to the tumor region to activate the gold nanoparticles, leading to a transient disruption of BBB integrity. This effect is thought to occur through an increase in paracellular permeability, likely mediated by calcium influx (Figure 5). As a result, drug-loaded nanoparticles are able to cross the BBB, enter the brain parenchyma, and achieve higher drug concentrations within the tumor [88]. In the study by Cai et al., (2024) the optoBBTB approach reduced tumor volume by 6-fold and 2.4-fold and extended survival by 50% and 33%, respectively [89]. Given that paclitaxel does not cross the blood–brain tumor barrier and has been abandoned for the treatment of glioblastoma following its lack of efficacy in early-phase clinical trials, these findings raise the possibility of reassessing a number of potent anticancer agents by combining them with strategies designed to enhance blood–brain tumor barrier permeability. Overall, this method offers several advantages, including regional specificity, which enables selective enhancement of drug delivery to the tumor while sparing the surrounding healthy brain tissue; reversibility, as blood–brain barrier integrity is restored within one day; and repeatability, allowing multiple cycles of chemotherapy to be combined with BBB modulation and targeted drug delivery to the tumor. Later, the same research group, Cai et al., confirmed the efficacy of this method using two clinically relevant genetically engineered mouse models of glioblastoma [89]. Regarding the very early stage of development of this method, no derivative approaches or studies have been conducted to date, including applications for the delivery of miRNA-based therapeutics.

**Table 3:** Resume of current locoregional delivery methods.

Locoregional Delivery Method	Advantages	Disadvantages	Refs.
CED	CED expands intratumoral drug distribution using pressure gradients for uniform delivery over large volumes; Enables delivery of larger therapeutic molecules; Covers larger tissue areas in shorter treatment durations; Prolongs drug retention time, enabling sustained release in targeted tissues; Limits the associated systemic toxicities.	Invasive; As CED requires the placement of a cannula, it is more invasive; Variability in surgical methods and catheter placement impacts reliability; Complexities in drug delivery due to backflow, air bubbles, and tissue resistance; Challenges in achieving optimal drug concentrations due to white matter edema, tumor heterogeneity, and the ratio of infusion volume to distribution volume; Differences in the mechanisms of action of drugs make standardizing treatments difficult; Limits comprehensive clinical trials and broader usage due to high financial burden.	[91,92]
Intranasal drug delivery	Non-invasive; The avoidance of the systemic circulation, reducing the risk of systemic side effects and hepatic/renal clearing; The possibility of chronic administration; The self-administration by patients; The rapid onset of action.	The rapid removal of a drug from the nasal cavity by ciliated cells; Efflux mechanisms involving transport proteins expressed in the nasal mucosa can limit the absorption of certain drugs; Nasal cavity enzymes can destroy some drugs; Capillaries in the nasal cavity can adsorb drugs in the general circulation.	[93,94]
Intrathecal injection	Relative consistency in the surgical procedure; Large target volume to CSF without reaching brain parenchyma; No postoperative recovery period is required.	Invasive; Risk of causing damage when the needle passes through the healthy brain tissue; There is a possibility for backflow of the drug; Asymmetry in drug distribution when only one ventricle is infused; Difficulty with repeat infusions without catheter assistance; Large injection volumes may create transient pressure gradients, potentially disrupting structures and impacting convective deliveries; The procedure is painful.	[48]
Spray-based delivery	May overcome current limitations in GBM treatment, such as the lack of therapeutic drug concentrations reaching residual GBM cells following surgery	Applicable only invasively, after surgery; not studied enough	[57]
Polymeric hydrogel system	Physical hydrogels are cost-effective, easily synthesized under mild conditions, and offer adaptability and precise control through dynamic swelling and adjustable crosslinking; Natural polymers ensure non-toxicity, biodegradability, and minimized immune rejection; Conformational changes in response to stimuli enable sustained and controlled drug release; Chemically synthesized hydrogels have high mechanical strength with good stability and tunability.	Must be implanted in brain—invasive; If applied with injection, there are problems with BBB; Physical hydrogels have reduced mechanical strength and lower stability issues due to weaker crosslinking interactions; Fine tuning of structure is hindered as precise cross linking cannot be achieved; Chemically synthesized hydrogels require complex preparation and are potentially toxic with poor reversibility.	[64,95]
FUS	Non-invasive Not toxic for health cells	Sometimes skin burns due to arbitrarily changing positions; Sometimes low-grade fever after treatment; The tissue surrounding the treated area may be affected by the ultrasound waves.	[74]
optoBBTB	Non-invasive Not toxic for health cells Reversibility of BBB	Less study at this moment—still largely experimental; The tissue may be affected by the laser; Limited light penetration—light (especially visible wavelengths) does not penetrate deeply into brain tissue; Tumor heterogeneity issues—brain tumors (especially glioblastoma) have heterogeneous vasculature, meaning: uneven barrier opening, inconsistent drug delivery across the tumor.	[87,88,89]

## 3. Discussion

Despite the promise of nanoparticle-based therapies for glioblastoma, their clinical translation remains limited. In clinical practice, treatment efficacy is constrained by tumor heterogeneity, diffuse infiltration, and poor drug penetration across the blood–brain barrier, with only modest survival benefit from standard therapies such as temozolomide. Multiple barriers contribute to this limited success, including biological constraints such as extracellular matrix (ECM) entrapment and sequestration by tumor-associated macrophages, as well as technical challenges related to reproducibility, large-scale production, and nanoparticle stability. Clinical and regulatory issues, including dose optimization, route of administration, and the lack of predictive biomarkers, further hinder translation. This is exemplified by a Phase I trial led by Andrew J. Brenner, where rhenium-186 nanoliposomes delivered via convection-enhanced delivery achieved favorable safety profiles and encouraging survival outcomes [96], yet therapeutic efficacy remained strongly dependent on dose distribution and tumor coverage. These findings underscore delivery efficiency as a major bottleneck.

In this context, a wide range of locoregional approaches—including convection-enhanced delivery, implantable drug-eluting systems, focused ultrasound-mediated barrier disruption, and emerging optical and nanotechnology-based methods—have demonstrated the capacity to improve intratumoral drug delivery within tumors in many preclinical animal models (Table 3). These strategies represent a rational approach to circumvent the major limitations of systemic therapies, notably the restricted permeability of the blood–brain barrier and the nonspecific distribution of intravenously administered agents. Moreover, these approaches offer an opportunity for the direct administration of drugs into the tumor cavity, enabling the achievement of high pharmacologically active concentrations at the disease site while minimizing systemic toxicity. Of particular interest, sustained-release platforms—including biodegradable polymers, hydrogels, and implantable depots—can prolong postoperative drug delivery by targeting residual tumor cells over an extended period of time, which is especially relevant in glioblastoma, where local recurrence remains the predominant cause of treatment failure. However, despite a strong biological rationale and consistent preclinical efficacy, the clinical benefit of locoregional delivery is still modest. Most locoregional strategies necessitate surgical interventions—such as catheter placement, device implantation, or intraparenchymal injection—which are invasive, cause patient discomfort, and limit the feasibility of repeated administrations and long-term disease control. Therefore, in this context, recent delivery methods such as focused ultrasound (FUS) and optical blood–brain tumor barrier (optoBBTB) techniques are of considerable interest, as they offer alternatives to invasive surgical implantation of catheters or devices at the tumor site. Both approaches are minimally to non-invasive. The FUS method employs systemic microbubbles combined with localized ultrasound to transiently open the blood–brain barrier (see Section 2.7), whereas optoBBTB uses near-infrared light to activate systemically administered photosensitive agents to disrupt temporarily the tumor vasculature (see Section 2.8). These strategies enable systemic drug delivery while producing a locoregional effect that enhances tumor vascular permeability and improves passive drug penetration. Nevertheless, both methods remain relatively novel, and their ability to deliver precise amounts of drug or payload to the tumor site has not been fully characterized. Further studies are required to comprehensively assess their efficacy and therapeutic potential. In addition, both intra- and intertumoral heterogeneity within glioblastoma tumor masses—which evolves dynamically over the course of tumor development—limits the efficacy of currently administered drugs, particularly first-line chemotherapeutic agents such as temozolomide. The efficacy of locoregional delivery is also inherently limited by the diffuse and infiltrative nature of glioblastoma. These tumor cells frequently extend beyond resection margins and treatment fields. As a consequence, it is difficult to achieve homogeneous and predictable intraparenchymal distribution. In addition, elevated interstitial fluid pressure, extracellular matrix heterogeneity, and white-matter anisotropy contribute to uneven drug dispersion, subtherapeutic regional concentrations, and interpatient variability. These constraints are particularly critical for convection-based and injectable systems, where subtle variations in catheter positioning or infusion parameters can markedly influence therapeutic outcomes [97].

Over the past two to three decades, considerable efforts have been directed toward addressing the limited efficacy of conventional chemotherapeutics through the development of therapies with broader activity spectra and multiple mechanisms of action. The main objectives are to circumvent tumor drug resistance, such as that observed with temozolomide [98], potentiate the therapeutic efficacy of chemotherapeutic agents through synergistic intervention, and to activate alternative cell death pathways. Several biotherapy agents have been evaluated for locoregional delivery in glioblastoma, including, but beyond out of scope of this review, oncolytic viruses (e.g., G47Δ, DNX 2401, PVSRIPO [99]; CAR T cells targeting tumor-associated antigens (e.g., IL 13Rα2, EGFRvIII, HER2; [55]), and stem cell-based delivery platforms [100].

Among oligonucleotide-based therapeutic biotherapy agents, miRNAs represent a promising class of molecules capable of modulating gene expression at the post-transcriptional level. As discussed in this review, several miRNAs have been extensively investigated as therapeutic agents in glioblastoma. Theses includes miR-34a, miR-124, miR-128, and miR-21 inhibitors, which regulate key oncogenic pathways such as apoptosis, proliferation, stemness, and invasion. These miRNAs are exploited either to restore tumor-suppressive functions (e.g., miR-34a, miR-124, miR-128) or to inhibit oncogenic miRNAs (e.g., anti-miR-21), offering a multifaceted approach to overcome tumor resistance and enhance the efficacy of standard therapies.

Several additional miRNAs have been identified as promising therapeutic targets in glioblastoma, including miR-7, miR-181, miR-302-367, miR-93, miR-196, miR-221/222, miR-296, and miR-125b [101]. Although most of them have been studied via alternative administration routes—such as subcutaneous xenografts with intratumoral injections, systemic delivery, or lentiviral-modified cell implantation—they are considered robust candidates for further investigation using locoregional delivery methods. For instance, miR-7 acts as a tumor suppressor following systemic delivery [102], while miR-181 has been delivered via hyaluronan-decorated lipid nanoparticles both intratumorally and systemically [103,104], resulting in improved median survival in glioblastoma-bearing mice. The miR-302-367 cluster has been explored through exosome-mediated delivery in intracranial models [105], whereas miR-93 and miR-125b have primarily been studied via lentiviral modulation in intracranial models [106,107]. Intratumoral delivery of antagomiR-196a reduces tumor proliferation [108], while antisense inhibition of miR-221/222 has been shown to induce apoptosis and significantly decrease tumor growth in xenograft models [109]. Advanced polymeric nanocarriers were also found to enable intratumoral delivery of miR-296-5p and miR-148a throughout the entire tumor mass, correlating with reduced tumor growth and increased sensitivity to γ-radiation in orthotopic glioblastoma xenograft models [110]. In addition, multi-targeting strategies involving multiple miRNAs have demonstrated better outcomes than single-miRNA approaches; however, this strategy remains relatively underexplored to date. For example, combinations of miR-17-3p, miR-222, and miR-340 significantly delay tumor growth, reduce proliferation, and enhance apoptosis [111]. Co-delivery of multiple miRNAs can be achieved using a single polycistronic plasmid incorporated into engineered exosomes (eExos), enabling administration via locoregional routes and resulting in prolonged survival of glioblastoma-bearing mice compared with eExos carrying each miRNA individually [112]. In contrast to miRNAs, siRNAs are highly sequence-specific, targeting a single mRNA, which enables predictable silencing and has facilitated clinical approval in liver-targeted diseases. Several siRNAs have been explored in glioblastoma therapy to selectively silence key oncogenes, including EGFR, VEGF, STAT3, PIK3R3, and β-catenin, which regulate proliferation, survival, angiogenesis, and therapy resistance [113,114,115,116]. To date, siRNAs are considered more “druggable” than miRNAs, with several siRNA-based therapeutics already in the clinic [117]. Consequently, over the past two to three decades, extensive research on siRNAs has generated a wealth of knowledge, including well-characterized sequence specificity, predictable pharmacokinetics, and optimized delivery platforms. It can be anticipated that miRNA-based therapeutics will benefit from these advances and are poised to achieve substantial progress in the coming years. Overall, a major advantage of miRNAs in cancer therapy is their ability to simultaneously regulate multiple genes within key oncogenic pathways. A carefully selected miRNA can therefore act as a master regulator of gene regulatory networks, modulating tumor proliferation, apoptosis, invasion, and resistance mechanisms in a coordinated manner. This multi-targeted activity allows miRNAs to offer unique therapeutic opportunities in multifactorial diseases such as cancer, where single-gene silencing is insufficient.

In light of the evidence discussed in this review, it becomes apparent that future research efforts should prioritize the rational optimization of delivery platforms, with particular emphasis on enhancing molecular stability, improving pharmacokinetic profiles, and increasing targeting specificity of therapeutic agents intended for combinatorial strategies. The successful implementation of such combination therapy will require the integration of multifactorial and highly complex datasets that remain challenging to collect. In particular, comprehensive knowledge of the expression of molecular targets is essential and should ideally be captured using noninvasive monitoring methods suitable for longitudinal analysis. In parallel, the development of advanced delivery platforms also represents a mandatory prerequisite. These systems must be capable of co-encapsulating and transporting at least two types of therapeutic agents, which are likely to differ substantially in their molecular composition, stability profiles, and physicochemical properties. This necessitates sophisticated carrier engineering to ensure structural compatibility, preservation of biological activity, controlled release kinetics, and optimal biodistribution of each component within the combination regimen. Ideally, these carriers should be compatible with locoregional delivery approaches, enabling repeated administration while minimizing toxicity and patient discomfort, thereby ensuring a sustained therapeutic effect. Finally, precise optimization of dosing regimens is also a critical parameter. This includes defining the appropriate dose ratios, scheduling, and sequence of administration, whatever drugs are delivered together or sequentially, while limiting adverse events. Ultimately, these strategies must also demonstrate cost-effectiveness to enable widespread clinical translation at minimal cost. Collectively, addressing these challenges represents a decisive step toward the advancement of precise molecular medicine.

## 4. Conclusions

In conclusion, locoregional delivery for glioblastoma offers significant practical advantages by circumventing systemic circulation and the blood–brain barrier, while enabling higher local concentrations of therapeutic agents at the tumor site. Despite these benefits, current limitations indicate that its clinical success has not yet been fully realized, although it is anticipated in the near future. Continued efforts are required to develop biocompatible, multifunctional delivery platforms capable of supporting repeated and longitudinal administration of multiple therapeutics—either alone or in combination—while minimizing brain toxicity and patient discomfort. Future progress is likely to depend on combinatorial strategies that integrate locoregional delivery with synergistic modalities, such as radiotherapy, targeted molecular agents, and immune checkpoint inhibitors, alongside advancements in biomaterials, real-time imaging technologies, and patient stratification based on molecular target expression. The locoregional administration of tumor-suppressive miRNAs and inhibitors of oncogenic miRNAs has demonstrated feasibility and efficacy in modulating multiple signaling pathways, offering a substantial advantage over conventional siRNA-based approaches, and highlighting their potential for early clinical translation. Overall, such integrated and adaptive strategies have the potential to fully unlock the therapeutic promise of locoregional delivery within multimodal glioblastoma treatment.

## Figures and Tables

**Table 1:** CED of MicroRNA-based Therapeutics in In Vivo Glioblastoma Models at preclinical stage.

Year	MicroRNA-Based Therapeutic Agents	RNAi Activity	Therapeutic Efficiency at Preclinical Stage	Refs.
2015	anti-let-7	Alterations in mRNA levels have been shown to account for ≥84% of the altered target protein level, upon miR inhibition.	Not evaluated	[26]
2016	anti-miR-10b	The efficacy of miR-10b inhibition in intracranial tumors was assessed by qRT–PCR analysis of resected tumor tissues. Reported data indicated around 92% of knockdown of miR-10b expression in tumor tissues. qRT–PCR analysis further demonstrated that the miR-10b target gene p21 was de-repressed in GL261 tumors following miR-10b inhibition. mRNA expression levels were normalized to GAPDH. Relative mRNA levels were approximately 4 in the control group and around 7 in miR-10b inhibitor-treated tumors. Alterations in mRNA levels have been shown to account for around 43% of the altered target protein level, upon miR inhibition.	Continuous delivery of miR-10b ASO for 2 weeks significantly reduced the progression of intracranial GBM. In the treated group, the tumor size was approximately 60% smaller.	[29]
2019	anti-miR-21	PCR analysis indicated that treatment with PACE-anti-miR, PLA-HPG, or PLA-HPG-CHO NPs resulted in 67%, 53% and 49% knockdown of miR- 21 expression compared to untreated animals.	PACE-anti-miR NPs with TMZ—108% increase in survival compared to PBS control; PLA-HPG-CHO NPs and TMZ—104% increase in survival compared to PBS control;	[30]
2020	miRNA-133 and -200c	Approved with miRNA-ON bioluminescence imaging system—RILES (RNAi-inducible expression Luciferase system)	Evaluated for miR 200c—7% increase in survival compared to control (*p* < 0.05)	[32]
2021	miRNA-133a	Approved with miRNA-ON bioluminescence imaging system—RILES (RNAi-inducible expression Luciferase system)	Not evaluated	[33]
2023	sγPNAs, targeting the oncomiRs 10b and 21	**72%** knockdown of miR-10b and 95% decrease in miR-21 expression	sγPNA/BNP combined with TMZ—135% increase in survival compared to control	[34]

**Table 2:** Intranasal Drug Delivery of MicroRNA-based Therapeutics in In Vivo Glioblastoma Models at preclinical stage.

Year	MicroRNA-Based Therapeutic Agents	Vector	RNAi Activity	Therapeutic Efficiency at Preclinical Stage	Refs.
2019	anti-miR-21, miR-100	targeted polyfunctional gold–iron oxide nanoparticles	PCR analysis showed significant knockdown (around 88%) of miR-21 expression compared to untreated control	miR-100-AmiR-21 + TMZ- > 250% increase in survival compared to control	[44]
2021	antagomir-21, RAGE-antagonist peptide (RAP)	self-assembled antagomir-21/RAP nanoparticles	PCR analysis showed 50–60% knockdown of miR-21 expression compared to untreated control	antagomir-21, RAGE-antagonist peptide (RAP)—10–20% increase in survival compared to control	[45]
2021	anti-miR-21, miR-100	CXCR4-engineered extracellular vesicles	PCR analysis showed 80% knockdown of miR-21 expression compared to untreated control	anti-miR-21, miR-100 + TMZ—130% increase in survival compared to control	[46]

## Data Availability

No new data were created or analyzed in this study. Data sharing is not applicable to this article.
